# Exploitation of an ancestral pheromone biosynthetic pathway contributes to diversification in *Heliconius* butterflies

**DOI:** 10.1098/rspb.2022.0474

**Published:** 2022-07-27

**Authors:** Bruna Cama, Stephanie Ehlers, Daiane Szczerbowski, Jane Thomas-Oates, Chris D. Jiggins, Stefan Schulz, W. Owen McMillan, Kanchon K. Dasmahapatra

**Affiliations:** ^1^ Department of Biology, University of York, Heslington YO10 5DD, UK; ^2^ Department of Chemistry, University of York, Heslington YO10 5DD, UK; ^3^ Institute of Organic Chemistry, Technische Universität Braunschweig, Hagenring 30, Braunschweig 38106, Germany; ^4^ Department of Zoology, University of Cambridge, Downing Street, Cambridge CB2 3EJ, UK; ^5^ Smithsonian Tropical Research Institute, Balboa, Ancón, Panama

**Keywords:** macroevolution, sympatric speciation, reproductive isolation, chemical ecology

## Abstract

During courtship, male butterflies of many species produce androconial secretions containing male sex pheromones (MSPs) that communicate species identity and affect female choice. MSPs are thus likely candidates as reproductive barriers, yet their role in speciation remains poorly studied. Although *Heliconius* butterflies are a model system in speciation, their MSPs have not been investigated from a macroevolutionary perspective. We use GC/MS to characterize male androconial secretions in 33 of the 69 species in the Heliconiini tribe. We found these blends to be species-specific, consistent with a role in reproductive isolation. We detected a burst in blend diversification rate at the most speciose genus, *Heliconius*; a consequence of *Heliconius* and *Eueides* species using a fatty acid (FA) metabolic pathway to unlock more complex blends than basal Heliconiini species, whose secretions are dominated by plant-like metabolites. A comparison of 10 sister species pairs demonstrates a striking positive correlation between blend dissimilarity and range overlap, consistent with character displacement or reinforcement in sympatry. These results demonstrate for the first time that MSP diversification can promote reproductive isolation across this group of butterflies, showcasing how implementation of an ancestral trait, the co-option of the FA metabolic pathway for pheromone production, can facilitate rapid speciation.

## 1. Introduction

Invasion of a new ecological niche can lead to evolutionary radiation, leading to enhanced ecological or morphological disparity among the resulting species as they diverge to exploit different aspects of the niche [1]. This increased disparity can result from novel and unique evolutionary changes, often called key innovations, that accompany high diversification rate in the clade in which they are found [2]. These innovations are thought to facilitate diversification by unlocking ‘ecological opportunity’: the ability for species to diverge into underused ecological niches [1,3,4]. Niche expansion and the resulting ecological opportunity can make room for new adaptations and in turn pave the way to an evolutionary radiation in a number of different ways [1,4]. In theory, proving that trait innovations are linked to diversification involves showing that the clade bearing the innovation has undergone faster diversification than its relatives, that such diversification has occurred in tandem with the evolution of a trait, and that the trait has also triggered adaptive phenotypical changes in the organism [4]. In practice, the difficulty is often with correctly identifying a trait as a key innovation, since an innovation such as a change in diet [5–7] is in reality a composite of multiple traits that have evolved simultaneously.

However, a novel trait does not need to be part of a key innovation to open up new niches at a finer level. The recovery of a previously lost trait, while by definition not an innovation, may also have a similar effect, and testing its effect on clade diversification may pose fewer issues than larger changes that arise with the modification of multiple traits. Niches are often thought of in an ecological sense, as the range of different positions occupied by organisms within the ecosystem, such as the variety of food sources, feeding substrates or habitat preferences [8]. But with traits involved in reproductive isolation, niches can reflect the availability of signal space. Reproductive character displacement may lead to the evolution of different mating signals that do not overlap with those of closely related species [9,10]. The rapid acquisition of such non-overlapping signals is facilitated when the traits in question can evolve in a relatively unconstrained manner.

Chemical odours are important intra and interspecific signals across the tree of life, conveying information about fitness, reproductive status, species and individual identity, as well as serving as alarm or aggregation calls, among a variety of other purposes [11]. Pheromone signalling is a widespread form of chemical communication that elicit behavioural or physiological responses in receiving individuals [12–14]. They are extremely variable among species, both in function and in composition, and are an essential communication system in unicellular and multicellular organisms [15]. Most macroevolutionary studies of signalling tend to focus on more easily quantifiable signalling, like traits involved in visual and auditory communication. Therefore, despite the ubiquity of pheromone signalling, the difficulty of testing and quantifying such signals and their behavioural effects means that pheromone macroevolution is a largely under-explored topic, though studies on the topic do exist, most commonly in insects [13,16,17]. While the role of pheromones in reproductive isolation has been explored before [18] there is a general lack of studies on the expansion and exploitation of odour niche space to facilitate reproductive isolation.

Neotropical *Heliconius* butterflies are representative of the bias towards visual traits. Over a century of research on this genus [19] has provided answers to many evolutionary questions pertaining to the role that the vivid, aposematic wing patterns that characterize the radiation play in adaptation and speciation [20–25], as well as adaptive introgression [21,23,26,27], and the role of ‘magic traits’ [28,29] in animal evolution. By contrast, relatively little attention has been paid to other traits involved in reproductive isolation, including the role of chemical signalling. Yet reproductive isolation among *Heliconius* species cannot be fully explained by colour pattern alone, and other ecological traits, including male sex pheromones (MSPs), also likely have key roles [29–31]. Nonetheless, from the early observations of Bates [32] on the possible role of mimicry on the increased rate of speciation in *Heliconius*, it took until 2008 for the first *Heliconius* pheromone to be identified [33].

MSPs are good candidates as reproductive barriers in butterflies due to their effect on mate choice. In *Heliconius*, these are produced by brush-like structures known as androconia located on the male hindwing (electronic supplementary material, figure S2) [34,35], and the chemical blends are mostly comprised of a mixture of fatty acid (FA) derivatives [35] and plant-like metabolites (including terpenes, aromatic compounds, and other chemicals commonly described in plants). In *Heliconius,* the plant-like metabolites may be acquired directly and/or transformed from plant products or they may be produced *de novo*, whereas the FA derivatives are synthesized via a single pathway and produced endogenously from ubiquitous FA precursors stored in the butterfly's body [35,36]. Both types of compounds are commonly found in lepidopteran pheromones [37]. The importance of these androconial compounds in mate choice is demonstrated by the fact that male *Heliconius* with blocked androconia are largely unsuccessful at mating compared to the untreated males [34], and at least one compound has been shown to have a function in mating through both behavioural and electrophysiological assays in *H. melpomene* [38]. Furthermore, in the co-mimetic sister species *H. melpomene* and *H. timareta,* females prefer males perfumed with conspecific rather than heterospecific androconial secretions [29], demonstrating the importance of scent in mating.

Rapid evolution of divergent pheromone blends may therefore be an important driver of reproductive isolation. The FA metabolic pathway, responsible for much of the androconial pheromone blend in *Heliconius* (and other Lepidoptera, including other species of nymphalid butterflies) [39,40], is modular in nature, comprising many reactions that can be activated or deactivated to produce different cocktails of final products [35] (electronic supplementary material, figure S1). This pathway thus has the potential to be evolutionarily labile, allowing species to rapidly occupy non-overlapping niches in odour signalling space. Use of the FA metabolic pathway may therefore have been instrumental in shaping the role of pheromones as reproductive barriers in this group of butterflies. Similar traits involved simultaneously in sexual signalling and mating isolation, such as courtship songs in *Drosophila,* have been shown to be particularly labile and able to diversify faster than traits not involved in mating [41].

Here we evaluate whether MSPs in the Heliconiini tribe of butterflies display macroevolutionary patterns consistent with those expected from a trait involved in reproductive isolation. We first determine the degree that MSP blends are species-specific. We then assess the rate at which pheromone blends evolve across this group of butterflies to determine whether species-rich clades have more rapidly evolving pheromone blends. Finally, we test the hypothesis that if pheromone blends are playing a key role in reproductive isolation then pheromone divergence between sister species should be correlated with the extent of geographical range overlap between them.

## 2. Methods

### (a) Gas chromatography/mass spectrometry analysis of androconial pheromones

**Figure 1.**
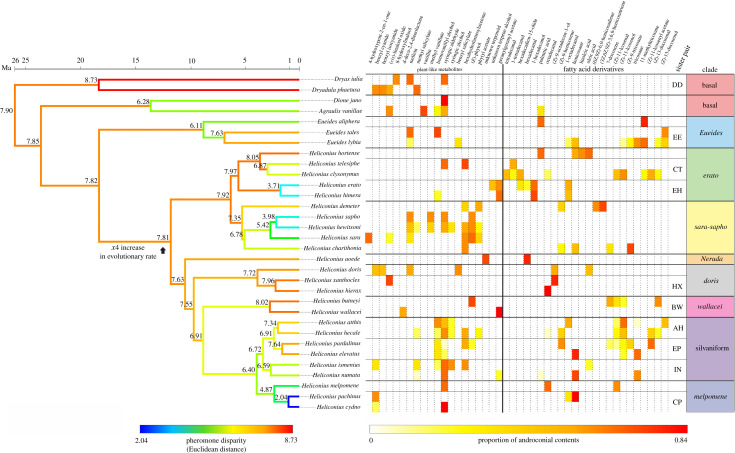
Heliconiini tree with branches coloured according to disparity in pheromone profiles. Values for disparity (expressed by Euclidean distance) are shown at each node. The table shows putative MSPs composition (Dataset C) of each species, where columns represent androconia-exclusive compounds and amounts are expressed as the proportion of the total androconial contents. Note the near absence FA derivatives among the basal genera, while in general *Eueides* and *Heliconius* species produce a varied cocktail comprising both plant-like metabolites and FA derivatives. This figure summarizes the most abundant compounds (at least 12% of the total androconial contents). For a complete dataset with all compounds, see (electronic supplementary material, table S6). (Online version in colour.)

We analysed the wing extracts of adult butterflies from 33 of the 69 Heliconiini species, obtained from either wild or captive-bred populations (figure 1; electronic supplementary material, table S2). From each species we analysed male extracts (1–10 individuals per species; 179 in total) and from 15 of these 33 species, we also analysed female extracts (1–7 individuals per species; 66 in total). Wild butterflies were sacrificed following capture, while captive-bred butterflies were sampled on reaching sexual maturity (greater than 10 days post-eclosion). Two wing tissues were dissected from males: the androconia, located along the forewing veins for the basal species and on the hindwings for *Eueides* and *Heliconius* [34,42,43]*,* and a control region of the wing not involved in pheromone secretion (electronic supplementary material, figure S2). The equivalent wing regions were sampled in females. Following dissection, the tissues were extracted using 200 µl of dichloromethane with a 2-acetoxytetradecane (1 ng µl^−1^) internal standard. Samples were then analysed on a gas chromatography/mass spectrometry (GC/MS) system as described in electronic supplementary material, Info S1.

AMDIS [44] was used to quantify and identify all compounds with gas chromatographic retention indices (RI) between 1031 (4-hydroxycyclopent-2-en-1-one) and 2900 (nonacosane) through comparison of mass spectra and RI values with synthetic samples and mass spectrometric databases (see [35] for full details of the GC/MS protocol and analysis). The known amount and molecular weight of the internal standard were used to calculate the amounts in nanomoles of all other compounds. Compound amounts were transformed to logged percentages (of the total amount of androconial contents) for use in most subsequent analyses.

We used these data to generate three datasets:

Dataset A: an unfiltered dataset comprising all compounds (1031 ≤ RI < 2600) detected in each individual with the exception of known contaminants and structural components (RI ≥ 2600).

Dataset B: a filtered subset of Dataset A comprising all compounds present in significantly different amounts in the androconia compared to the male control tissue in at least one species, as determined using one-tailed paired *t*-tests.

Dataset C: derived from Dataset B, this dataset retains for each species only the compounds present in significantly different amounts between androconia and male control tissues. We term these compounds putative MSPs. For *H. burneyi,* where only one male was sampled, we retained all compounds that only appeared in the androconia but not the control.

Most subsequent tests were run on both unfiltered and filtered datasets.

Female tissues were not used in the filtering due to the unavailability of female samples for many of the species included in the study; however, they were used for broad general comparisons between the contents of different tissues, shown in electronic supplementary material, table S3. The androconia for three additional taxa, *H. eleuchia, H. timareta* and *H. melpomene rosina,* were also available, but not accompanied by control samples, therefore these species were not included in any analysis post-filtering.

### (b) Pheromone diversity and species identity

For each species, using logged percentages of compounds for both the filtered dataset (Dataset B) and putative MSPs (Dataset C), we calculated the richness, evenness and diversity (Shannon Index) of androconial contents using vegan [45]. These statistics were compared between the basal and ‘advanced’ genera (*Heliconius* + *Eueides*) using Welch's two-sample *t*-tests. Differences in putative MSP (Dataset C) composition between species were visualized using non-metric multidimensional scaling (NMDS) on logged percentages. The NMDS was calculated on Euclidean distances using the metaMDS function in vegan, based on 1000 random starts and five dimensions. The number of dimensions (*k*) was chosen based on the stress level; five axes give an accurate representation of the data without overly inflating the number of dimensions.

To test whether pheromone blend is consistent within species as expected of a trait involved in reproductive isolation, we assessed the species-specificity of pheromone composition. Amounts from the filtered dataset (Dataset B) were converted into logged percentages, and pairwise Euclidean distances used as a measure of dissimilarity between individuals. Species-specificity was calculated using the ADONIS function (permutational analysis of variance based on distance matrices) from the vegan package, and significance was determined via 10 000 free permutations.

As a first step towards assessing the consistency of pheromone blend among related species, we also measured the clade-specificity of pheromone composition. The clades used are somewhat arbitrary but widely accepted [46], and are depicted in figure 1. Amounts from the filtered dataset (Dataset B) were averaged by species and converted into logged percentages. The Euclidean distance between species' average was used as a quantifier of dissimilarity. Clade specificity and significance were calculated as above.

### (c) Diversification rate of pheromone blends

We used the published ultrametric Bayesian tree for the Heliconiini [46] in all phylogenetic analyses. In species where samples from multiple races were available, a single race (*H. erato notabilis, H. melpomene plesseni* and *H. pardalinus butleri*) was chosen as representative of the species due to either good availability of samples or known branch length*.* This was to avoid the effect of polytomies, which can compromise the results of phylogenetic tests. We used the filtered dataset (Dataset B) for all the following phylogenetic analyses. The phylogenetic signal was quantified using the Kmult parameter [47] calculated with the physignal function in geomorph [48]. Significance was tested via 10 000 random permutations of the data between the tips of the phylogeny. To assess whether specific pathways or families of compounds were more phylogenetically conserved than others, Kmult was also calculated for the full blend as well as the following subsets of compounds: FA derivatives, plant-like metabolites and two families of plant-like metabolites separately: lignin-derived phenolic aromatic compounds and phytol-derived compounds. The former two were chosen due to their different biosynthetic origins, whereas the latter two were chosen as the two largest and most widespread groups of plant-like metabolites in the dataset.

Using mvMORPH [49], alternative evolutionary models were fitted to test different tempos of androconial compound evolution, as well as shifts in the rate of chemical blend evolution across the Heliconiini. The data used for this consisted of five-dimensional NMDS scores averaged within each species and based on Dataset B. The models tested include three non-shift models (Brownian motion, early burst and Ornstein–Uhlenbeck, also known as evolutionary constraint) as well as models with rate or mode shifts at two different points in the phylogeny: *Eueides* + *Heliconius*, and *Heliconius* (table 1). The reasons for these choices were as follows: the *Eueides/Heliconius* split is the base of the most diverse genera, and corresponds to the renewed implementation of the FA metabolic pathway for pheromone production as found in this study; the base of *Heliconius* marks the beginning of the *Heliconius* evolutionary radiation. The models were compared using the corrected Akaike information criterion (AICc) and the relative Akaike weight. The compare.evol.rates function from geomorph was then used on Dataset B as well as on its five NMDS axes to assess the shift in rate for the best fitting model, based on 10 000 random permutations.

**Table 1.** Evolutionary models of Heliconiini male sex pheromone diversification ordered from best (top row) to worst fit (bottom row). The best fitting model starts with Brownian motion followed by a burst of diversification at the root of *Heliconius*. The corrected Akaike information criterion (AICc) was used to assess model fit, and AICw (AIC weight) represents the likelihood of a particular model being the best fitting one among the six tested models.

model description	AICc	AIC diff	AICw	mode	N shifts	shift location
Brownian to early burst	603	0	1.00	BM-EB	1	*Heliconius*
Brownian to early burst	607	3.56	0.17	BM-EB	1	*Eueides–Heliconius*
Brownian with a rate shift	611	7.42	0.02	BMM	1	*Heliconius*
Brownian	622	18.57	0.00	BM	0	—
early burst	624	21.23	0.00	EB	0	—
evolutionary constraint	629	25.53	0.00	OU	0	—

Shifts tested with mvMORPH were chosen based on *a priori* information on the clade's evolutionary history. We also used MOTMOT [50] to find rate shifts in an unguided manner using the function transformPhylo.ML with the ‘TraitMedusa2’ (tm2) model. The minimum clade size for inferred rate shifts was set as 3 to avoid a bias for evolutionary shifts at the tips resulting from any strongly differentiated sister species pairs. The calcCutOff function was used to calculate an appropriate AICc cut-off for the tm2 model, based on 1000 simulations with the minimum clade size threshold taken into account. This AICc cut-off was used to determine the best fitting TraitMedusa2 output between the basic no-shift Brownian motion model and evolutionary rate shift models.

### (d) Comparison of sister species pairs

If pheromones act as reproductive barriers, sympatric sister species pairs are expected to produce more strongly divergent pheromone blends as a result of character displacement, compared to sister pairs with little or no range overlap [9,10]. We used a GLM with Gaussian distribution to test the effect of range overlap as reported in [51] and divergence times (branch length from [46] on pheromone dissimilarity (Euclidean distance, calculated using the unfiltered Dataset B with Geiger [52] between 10 of the 22 known Heliconiini sister species pairs, for which we had data [51]. Divergence times between sister pairs were included to control for evolutionary time.

### (e) Correlations between compounds

The co-occurrence of compounds across multiple species may arise simply as a consequence of compounds sharing the same biosynthetic pathway. Alternatively, such a correlation may be biologically more significant, such that two or more compounds act together to elicit a behavioural response. This was previously observed in *Heliconius melpomene* anti-aphrodisiac pheromones, where early eluting volatile compounds and heavier late eluting FA esters had a combined behavioural effect that was stronger than isolated early volatiles and esters [33]. Using species averages for each compound, we tested for correlations between all pairs of compounds in the filtered dataset (Dataset B). To correct for the phylogenetic relatedness, we computed the phylogenetic variance–covariance matrix and then converted it into a correlation matrix, which was then visualized using corrplot [53]. We calculated the *p*-values separately as described in [54]. Due to the large number of correlation tests resulting from this analysis, we used the Benjamini–Hochberg procedure to correct *p*-values and control for the false discovery rate [55] using the p.adjust function from the R base stats package.

## 3. Results

GC/MS analyses detected 127 compounds from 231 samples (170 males and 61 females) representing 36 Heliconiini species, three of which (*H. erato, H. melpomene* and *H. pardalinus*) included two races. From these, we removed cuticular hydrocarbons (RI ≥ 2600) as they are ubiquitous in insect tissues and too heavy for longer range signalling. All of these compounds were long chain alkanes, and there were only five instances where a cuticular hydrocarbon was significantly higher in proportion in the androconia compared to the control: hexacosane in *D. juno,* octacosane in *D. phaetusa* and *H. pachinus,* and nonacosane in *E. tales.* Owing to the ubiquity of cuticular hydrocarbons, these differences are likely due to different amounts of sampled tissue. This left us with 117 compounds, making up Dataset A (electronic supplementary material, table S7). Three taxa, *H. eleuchia, H. melpomene rosina* and *H. timareta,* lacked control samples and were thus not included in any filtering stage of the analysis, leaving a total of 33 species. Only 87 of the 117 compounds were significantly higher in proportion in the androconia compared to the male control in at least one species, as per Welch's two-sample *t*-test. These 87 compounds were used in the filtered analysis, making up Dataset B. The same 87 compounds make up Dataset C. For Dataset C, however, we only retained, for each individual, compounds present in significantly higher amounts in the androconia of that species compared to the negative control, while nullifying the amounts of other compounds that do not show that significant difference. We refer to the retained compounds for each species as ‘putative MSPs’, as the behavioural activity of most of them has not been ascertained. In most species, male androconia samples contained significantly more compounds, and in significantly larger quantities than both male hindwing and female controls (electronic supplementary material, table S3), demonstrating the role of androconia as male tissues specialized for secreting complex chemical blends [31] This is consistent with behavioural studies in several *Heliconius* species which have shown that compared to unmanipulated males, males whose androconial output has been blocked have strongly reduced mating success [34]. For a list of all 117 compounds and information on which were retained in Datasets B and C, see electronic supplementary material, table S7.

### (a) Qualitative comparison of advanced and basal Heliconiini

The androconia extracts of the advanced Heliconiini (*Heliconius* + *Eueides*) contained significantly more compounds than those of the basal Heliconiini (*Philaethria*/*Dione*/*Dryadula*/*Dryas*/*Agraulis*); on average 20 ± 7.6(SD) versus 11 ± 3.1(SD) compounds, respectively (Welch's two-sample *t*-test, *t* = 4.36, d.f. = 9, *p* = 0.002) when considering Dataset A. In Datasets B and C, this pattern remains unchanged, with 17 ± 6.3(SD) versus 10 ± 2.9(SD) compounds respectively (*t* = 4.13, d.f. = 8, *p* = 0.004) for B and 7 ± 4.2(SD) versus 3 ± 1.9(SD) compounds respectively (*t* = 3.09, d.f. = 8, *p* = 0.01) for C. The evenness of compounds was not significantly different between the advanced and basal Heliconiini regardless of the filtering level (Dataset B: *t* = −1.30, d.f. = 4, *p* = 0.27; Dataset A: *t* = −2.67, d.f. = 4, *p* = 0.08). Likewise, the diversity (Shannon index) of compounds in the androconial extracts was not significantly different between the two groups in any dataset (Dataset B: *t* = 1.87, d.f. = 5, *p* = 0.12; Dataset A: *t* = 1.34, d.f. = 4, *p* = 0.26). Therefore, while the number of constituent compounds of the advanced Heliconiini pheromone blend is greater than that of the basal Heliconiini, the distribution of such compounds, as expressed by the evenness, is similar between the groups, resulting in similar diversity indices. These results are summarized in electronic supplementary material, table S4.

An examination of the putative MSPs (present at significantly higher amounts in the androconia compared to the control tissues) of the Heliconiini reveals a major difference in the pheromonal contents of basal and *Heliconius/Eueides* when it comes to the two broad categories of compounds found in these species: plant-like metabolites and FA derivatives. We refer to all non-FA-derived compounds as plant-like metabolites, as they have all been previously described in plants, although we are not certain whether they are all acquired from plants or other exogenous sources, and lepidopterans are capable of producing plant-like products themselves [56–58]. The compound blends of *Heliconius* and *Eueides* species usually include a mixture of FA and plant-like metabolites, with the only exception being *H. hierax* (whose only putative MSP is octadecanal, a FA derivative) and most analysed species in the *sara-sapho* clade of *Heliconius* (whose putative MSPs are all plant-like metabolites) (figure 1). By contrast, the putative MSPs of the basal Heliconiini comprise only plant-like compounds, except for one FA (palmitic acid) found in larger amounts in the androconia of *Agraulis vanillae.* This indicates that while all Heliconiini species are able to produce FA derivatives, the ability within this tribe to metabolize increased amounts of FA derivatives as part of the androconial blend of compounds is an acquired trait in *Heliconius* and *Eueides*. The basal Heliconiini blend, with its lack of these compounds, appears rather atypical given how widespread FA derivatives are in pheromones blends across lepidopterans [59–61], and in fact the closely related *Cethosia cyane* is able to synthesize a variety of FA derivatives in its pheromone-producing tissues [62]. In this sense, FA derivative-inclusive blends can be seen as an ancestral lepidopteran trait [40] which, in the context of pheromone production, was lost between *Cethosia* and the basal Heliconiini and subsequently regained in *Eueides–Heliconius*.

Despite the variety of blends observed in the Heliconiini, species remains a very strong predictor of pheromone composition (ADONIS, *R*^2^ = 0.85, *p* < 0.0001), meaning that androconial contents tend to not vary much within a species. In contrast to species identity, clade identity explains much less variation in pheromone composition (ADONIS, *R*^2^ = 0.35, *p* < 0.0001). Thus, while there is a tendency of related species to have similar androconial blends, this does not explain much of the diversity seen in the data. These patterns can be visualized in the NMDS plot based on Euclidean distance (reflecting the dissimilarity values used by the ADONIS test) in (electronic supplementary material, figure S3).

### (b) Phylogenetic analysis

Tests on the phylogenetic signal of the full blend on Dataset B returned a low value of Kmult (*K* = 0.3). Similarly low but significant phylogenetic signal was found for most subsets of the data investigated, including FA derivatives, plant-like metabolites and phenolic (lignine) derivatives, with the only category not returning a significant signal being phytol derivatives (electronic supplementary material, table S5). While this result is discordant with the near absence of phylogenetic signal in *Heliconius* sex pheromones reported previously [35], the difference is likely due to the more comprehensive sampling of species reported here (33 versus 11 species analysed in [35] (electronic supplementary material, table S1A). Out of all the evolutionary models tested with mvMORPH, the data are best explained by a Brownian motion to early burst (BM-EB) mixed model with the mode shift at the root of *Heliconius* (table 1). Based on the corrected AIC weights, this model is 5.8 times (AICw_heliBMEB_/AICw_advBMEB_ = 1/0.17) more likely than the next best model of BM-EB with the shift at the root of (*Eueides* + *Heliconius)* [63] (table 1). This means that a burst of diversification in MSP composition has accompanied the *Heliconius* radiation. Using the geomorph compare.evol.rates function, we find a significant approximately fivefold increase in rate between basal species and *Heliconius* and between *Eueides* and *Heliconius,* but no significant change between basal species and *Eueides*, a result which remains unchanged whether tested on the data itself or on the NMDS axes.

MOTMOT's unguided tm2 algorithm also finds a shift at the root of *Heliconius* with an approximately fourfold increase in evolutionary rate*,* corroborating what was found with *a priori* information via mvMORPH. However, statistically the rate shift model is not a better fit to the data compared to a simple Brownian model (ΔAICc = 17.36; AICc cut-off = 20.25).

### (c) Effect of sympatry on sister species

**Figure 2.**
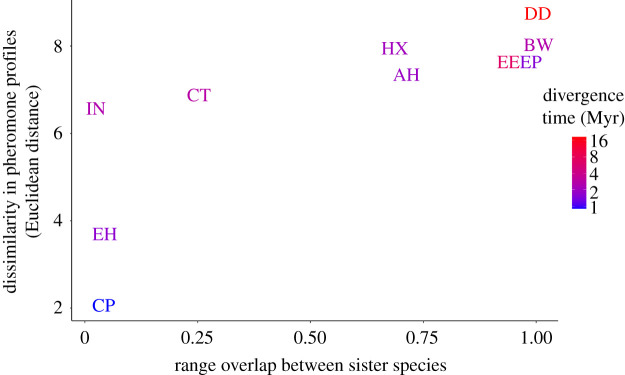
Relationship between dissimilarity in MSPs (Euclidean distance) and range overlap between Heliconiini sister species pairs. Divergence time between each sister species pair is represented as a logged colour scale. Each point is a pair of sister species: AH, *H. atthis–H. hecale*; BW, *H. burneyi–H. wallacei*; CP, *H. cydno–H. pachinus*; CT, *H. clysonimus–H. telesiphe*; DD, *Dryas iulia–Dryadula phaetusa*; EE, *Eueides tales–Eueides lybia*; EH, *H. erato–H. himera*; EP, *H. elevatus–H. pardalinus*; HX, *H. hierax–H. xanthocles*; IN, *H. ismenius–H. numata*. (Online version in colour.)

Based on the assumption that species discriminate conspecifics using differences in pheromone blends, we tested for evidence of character displacement by examining the relationship between the dissimilarity of pheromone blends between 10 Heliconiini sister species pairs and extent of range overlap, while taking account of divergence time. Our linear model, which explains 63% of the variance in pheromone dissimilarity (*R*^2^ = 0.63, *F*_7,9_ = 5.99, *p* = 0.03), shows a significant relationship between sister species pheromone dissimilarity and range overlap (GLM, *β* = 3.59, *t* = 2.85, *p* = 0.02), but not branch length (GLM, *β* = 0.05, *t* = 0.48, *p* = 0.64) (figure 2). Thus, range overlap rather than evolutionary distance strongly affects difference in pheromone composition between sister species pairs (figure 2). For a full table of dissimilarity values, branch lengths and range overlap, see electronic supplementary material, table S6.

### (d) Correlations among pheromone blend compounds

The correlation matrix (electronic supplementary material, figure S4) shows strong positive associations between several groups of compounds, among which the following are prominent: (i) a cluster of phenolic compounds (benzyl salicylate and phenylacetaldehyde) accompanied by their cyanide-containing relatives (benzyl cyanide and benzyl isocyanide phenylacetaldehyde oxime) and the unrelated (*Z*)-9-octadecen-1-ol, (ii) a subset of potential floral compounds (linalool, *cis*-linalool oxide in pyranoid form, and methyl salicylate) which may be synthesized by the butterflies themselves [56,57] alongside vanillin, (iii) several FAs, (iv) a cluster of straight chain and methylated alkanes, (v) several alcohols (including FA-derived alcohols as well as the plant-derived homovanillyl alcohol), (vi) phytol-related compounds (including (*E*)-phytol, (*E*)-phytal, phytyl acetate and its degradation product hexahydrofarnesylacetone), and (vii) phenolic compounds syringic alcohol, acetosyringone and syringic aldehyde. The presence of these clusters of correlated compounds suggests the possibility that compounds within each cluster may share biosynthetic pathways or in the case of chemically unrelated compounds, be required for more effective signalling.

## 4. Discussion

We show here that the evolutionarily labile FA metabolic pathway is not deployed in the androconial blends of basal Heliconiini. However, the pathway is reactivated in *Eueides* and *Heliconius* where it is associated with fast diversification of pheromones in the *Heliconius* radiation, in turn making character displacement of chemical signals possible in sympatric sister pairs in relatively short evolutionary timescales. The reactivation of this ubiquitous ancestral pathway seems to have provided the opportunity for niche invasion, with new niches represented by new signal space. Pheromone dissimilarity between sympatric pairs is dramatic, with the two species often having no major compounds in common, a testament to the rapidity with which these signals can evolve. These chemical blends show a high degree of species-specificity and usually very low within-species variation, both important properties of traits involved in assortative mating [18,64]. These results provide the strongest evidence to date of the role of MSPs as reproductive barriers across the *Heliconius* radiation.

### (a) Fatty acid derivatives-driven burst in the diversification of *Heliconius* pheromones

Phylogenetic analysis of our data suggests that factors other than neutral change [65] are responsible for the evolution of the wide variety of pheromone blends among the Heliconiini. A value of *K* < 1 indicates that the traits are more diversified among close relatives than they would be under random expectations [66,67]. With little dependence on their phylogenetic history, these traits could be considered evolutionarily labile and thus able to diversify rapidly, a pattern often seen in behavioural traits involved in intraspecific signalling such as bird songs [66]. In all cases, FA derivatives showed weaker phylogenetic signal than plant-like metabolites (electronic supplementary material, table S5), indicating that while both compound classes are evolutionarily labile, the former is relatively more flexible. The higher K value obtained when including only advanced species is more likely due to reduced resolution of the test, than to high diversity of the basal species.

We observe a burst in pheromone diversification rate at the root of *Heliconius*. Given the striking difference in androconial composition between the basal (predominantly plant-like metabolites) and *Eueides/Heliconius* (mixtures of FA and plant-like metabolites) species, it appears that usage of the FA metabolic pathway for pheromone production promoted this diversification. Obtaining the metabolic or physiological tools to invade new niche spaces is often accompanied by rapid diversification as seen in such examples as corallivory in *Chaetodon* butterflyfishes [5] or the shift to a nectarivorous diet in Hawaiian honeycreepers [7]. The *Heliconius* radiation itself is known to have probably been facilitated by a key innovation not observed in other Lepidoptera: pollen feeding, which brought about an array of new adaptations [6,68]. While the FA metabolic pathway's implementation constitutes a less dramatic physiological change, it still had an analogous effect in making new chemical sexual signalling tools available to *Heliconius* and *Eueides*.

Somewhat surprisingly, *Eueides* species use FA products in their pheromone blends yet do not show the same blend diversity as *Heliconius*. This may be an artefact of our dataset containing only three of the nine described species of *Eueides* [46]*.* It is plausible that with the inclusion of more *Eueides* species, the evolutionary shift may indeed be detected at the root of *Heliconius/Eueides*, rather than the root of just *Heliconius*.

In spite of their absence in the pheromone blends of the basal Heliconiini, FA-derived compounds are nearly ubiquitous in Lepidopteran pheromones [59–61], and there is some evidence that the biosynthetic pathways are conserved between moths and butterflies [39]. *Cethosia*, considered part of a sister group to the Heliconiini tribe [69], produces chemical signals that include both plant-like metabolites and FA derivatives [62]. This indicates that the FA pathway still retained its role in pheromone production at the time of the *Cethosia*–Heliconiini split. Therefore, FA-based pheromones appear to be an ancestral Lepidopteran trait, which was subsequently lost at the root of the Heliconiini and regained with the split of *Eueides/Heliconius* from the basal genera. However, while we were able to ascertain its derived status within the confines of the Heliconiini tribe, we currently lack pheromone data from enough species to make strong inferences about when losses and gains of this trait may have occurred in Lepidopteran evolutionary history, so whether usage of the FA pathway in pheromone production is an ancestral or derived condition across this insect order remains uncertain.

In addition to the clear differences in pheromone composition between basal and advanced species, with our comprehensive sampling of species we find that while no compound is exclusive to a single species, each species has its own unique blend of compounds, confirming previous work carried out on fewer species [35,70]. The high observed species-specificity of pheromone composition is consistent with the role of pheromones as potential reproductive barriers in these species, and it is partially achieved through the implementation of the FA pathway, making it one more consequence of its reactivation in *Eueides*/*Heliconius*. Traits involved in assortative mating are known to evolve under divergent selection [28,71] yet strong species-specificity of the mating cue is expected, especially in species with overlapping distributions [18,72], in order to obtain a response and provide reliable information to the receiving party [72].

### (b) Sympatry increases pheromone differentiation in sister species

Our findings that pheromone differentiation increases with the extent of range overlap between sister species provides by far the most compelling evidence that pheromone composition is important for reproductive isolation among Heliconiini species. This pattern of increased divergence between sympatric pairs is consistent with a scenario of reinforcement. Reinforcement is a phenomenon that may be observed when speciation occurs in complete or partial sympatry, where gene flow between the incompletely reproductively isolated taxa may promote the evolution of reproductive isolation because of selection for increased mate discrimination [73]. An important test of reinforcement is the relative strength of pre-zygotic reproductive barriers between sister taxa in different geographical contexts, with stronger barriers expected between sympatric taxa, with the potential for gene flow, than allopatric taxa [74]. This condition is unquestionably fulfilled in Heliconiini pheromones, where the strikingly divergent compositions seen in sympatric pairs would greatly improve discrimination in favour of conspecifics.

Since scent is a trait whose divergence reduces the likelihood of hybridization, pheromone divergence would be considered a case of reproductive character displacement [10,74,75]. Similar pheromone patterns are seen in *Bicyclus* [76], orchid bee pheromones [17] and *Hemileuca* moths [77], among others. They are however far from universally observed in sympatric species of insects [16], perhaps due to the varying importance of sex and aggregation pheromones in reproduction compared to other factors such as host plant or habitat preference.

Previous studies have highlighted that mate choice in many species of *Heliconius* butterflies is based on colour patterns, with males showing assortative mating based on visual cues [19,22,31,78]. However colour alone is not sufficient to explain male discrimination of heterospecific live females [22], or between closely related co-mimetic species [29], implying that more signals may be at play. Here we provide evidence that MSPs are important for initiating and/or maintaining species reproductive barriers across this group of butterflies.

There are two main implications to our findings. First, that the renewed exploitation of the FA metabolic pathway, not used in the pheromone-producing tissues of basal Heliconiini, accompanied the increased rate of diversification seen in this genus compared to other Heliconiini, meaning that it unlocked ecological opportunity. Second, that male androconial blend show macroevolutionary patterns expected of pre-zygotic reproductive barriers, with strong species-specificity, character displacement and the ability to diverge quickly in closely related species when needed, all of which minimize the chance of hybridization. The recruitment of a single family of enzymes, the Δ-11-desaturases, while themselves seemingly missing in *Heliconius* in favour of the widespread Δ-9-desaturases [35] preceded the Lepidopteran radiation, and is known to be key for pheromone diversification in the entire order, making numerous compound families available as pheromone components [61]. What happened with the FA pathway at the root of *Eueides*/*Heliconius* may be analogous to this, although at a smaller scale. It is not known whether pheromone diversification has been a primary driver for the iconic *Heliconius* radiation, perhaps concurrently with ecological differences such as habitat or host plant preferences, or whether it reinforced mating isolation following the diversification of other courtship cues such as the colour pattern. Nonetheless, we have detected, for the first time, strong evidence that chemical signalling cues have had an important part in the evolution and maintenance of the many *Heliconius* species seen today.

## Data Availability

All material pertaining to this publication is available through Dryad under accession https://doi.org/10.5061/dryad.51c59zw9w [79]. This includes the R code as well as all datasets needed to reproduce the statistical analyses presented here. The data are provided in electronic supplementary material [80].
